# Dichloridobis(phenanthridine-κ*N*)zinc(II)

**DOI:** 10.1107/S160053680901959X

**Published:** 2009-06-06

**Authors:** Zeinab Khoshtarkib, Amin Ebadi, Robabeh Alizadeh, Roya Ahmadi, Vahid Amani

**Affiliations:** a Islamic Azad University, Shahr-e-Rey Branch, Tehran, Iran; b Department of Chemistry, Islamic Azad University, Kazerun Branch, Kazerun, Fars, Iran; c Damghan University of Basic Sciences, School of Chemistry, Damghan, Iran

## Abstract

In the mol­ecule of the title compound, [ZnCl_2_(C_13_H_9_N)_2_], the Zn^II^ atom is four-coordinated in a distorted tetra­hedral configuration by two N atoms from two phenanthridine ligands and by two terminal Cl atoms. The dihedral angle between the planes of the phenanthridine ring systems is 69.92 (3)°. An intra­molecular C—H⋯Cl inter­action results in the formation of a planar five-membered ring, which is oriented at a dihedral angle of 8.32 (3)° with respect to the adjacent phenanthridine ring system. In the crystal structure, π–π contacts between the phenanthridine systems [centroid–centroid distances = 3.839 (2), 3.617 (1) and 3.682 (1) Å] may stabilize the structure. Two weak C—H⋯π inter­actions are also found.

## Related literature

For related structures, see: Ahmadi *et al.* (2008 ▶); Çelik *et al.* (2004 ▶); Cui *et al.* (1998 ▶); Gruia *et al.* (2007 ▶); Khalighi *et al.* (2008 ▶); Khan & Tuck (1984 ▶); Khavasi *et al.* (2008 ▶); Kozhevnikov *et al.* (2006 ▶); Liu *et al.* (2004 ▶); Markowitz *et al.* (2006 ▶); Musie *et al.* (2004 ▶); Preston & Kennard (1969 ▶); Reimann *et al.* (1966 ▶); Shen *et al.* (2004 ▶); Steffen & Palenik (1977 ▶). For bond-length data, see: Allen *et al.* (1987 ▶). 
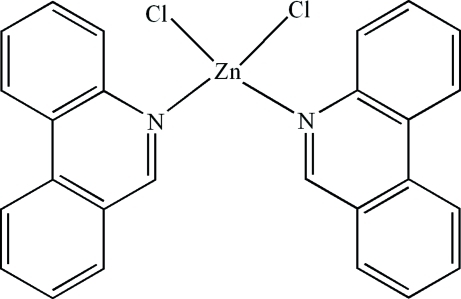


## Experimental

### 

#### Crystal data

[ZnCl_2_(C_13_H_9_N)_2_]
*M* *_r_* = 494.71
Monoclinic, 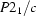

*a* = 16.193 (3) Å
*b* = 10.101 (2) Å
*c* = 14.491 (3) Å
β = 116.02 (3)°
*V* = 2130.0 (9) Å^3^
*Z* = 4
Mo *K*α radiation
μ = 1.42 mm^−1^
*T* = 298 K
0.45 × 0.30 × 0.22 mm

#### Data collection

Bruker SMART CCD area-detector diffractometer
Absorption correction: multi-scan (*SADABS*; Bruker, 1998 ▶) *T*_min_ = 0.610, *T*_max_ = 0.740
16947 measured reflections
5732 independent reflections
4612 reflections with *I* > 2σ(*I*)
*R*_int_ = 0.041

#### Refinement

*R*[*F* ^2^ > 2σ(*F* ^2^)] = 0.037
*wR*(*F* ^2^) = 0.086
*S* = 1.09
5732 reflections
280 parameters
H-atom parameters constrained
Δρ_max_ = 0.28 e Å^−3^
Δρ_min_ = −0.39 e Å^−3^

### 

Data collection: *SMART* (Bruker, 1998 ▶); cell refinement: *SAINT* (Bruker, 1998 ▶); data reduction: *SAINT*; program(s) used to solve structure: *SHELXTL* (Sheldrick, 2008 ▶); program(s) used to refine structure: *SHELXTL*; molecular graphics: *ORTEP-3 for Windows* (Farrugia, 1997 ▶); software used to prepare material for publication: *WinGX* (Farrugia, 1999 ▶).

## Supplementary Material

Crystal structure: contains datablocks I, global. DOI: 10.1107/S160053680901959X/hk2696sup1.cif

Structure factors: contains datablocks I. DOI: 10.1107/S160053680901959X/hk2696Isup2.hkl

Additional supplementary materials:  crystallographic information; 3D view; checkCIF report

## Figures and Tables

**Table:** 

Cl1—Zn1	2.2234 (7)
Cl2—Zn1	2.2456 (7)
N1—Zn1	2.0785 (17)
N2—Zn1	2.0775 (17)

**Table:** 

N2—Zn1—N1	105.19 (7)
N2—Zn1—Cl1	108.18 (5)
N1—Zn1—Cl1	106.23 (5)
N2—Zn1—Cl2	113.54 (5)
N1—Zn1—Cl2	107.46 (6)
Cl1—Zn1—Cl2	115.49 (3)

**Table 2:** Hydrogen-bond geometry (Å, °)

*D*—H⋯*A*	*D*—H	H⋯*A*	*D*⋯*A*	*D*—H⋯*A*
C1—H1⋯Cl1	0.93	2.77	3.434 (3)	129
C17—H17⋯*Cg*6^i^	0.93	2.82	3.535 (3)	134
C24—H24⋯*Cg*5^ii^	0.93	2.81	3.508 (3)	132

Symmetry codes: (i) 
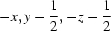
; (ii) 
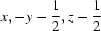
. *Cg*5 and *Cg*6 are the centroids of the C15–C20 and C21–C26 rings, respectively.

**Fig. 1.**
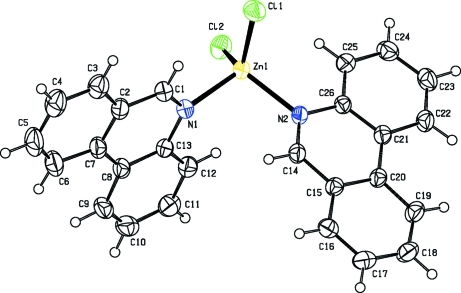
The molecular structure of the title molecule, with the atom-numbering scheme. Displacement ellipsoids are drawn at the 40% probability level.

**Fig. 2.**
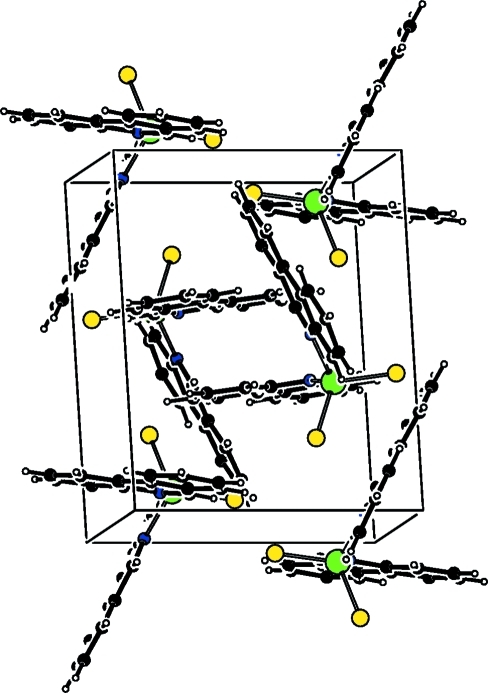
A partial packing diagram of the title compound.

## supplementary crystallographic information

### Comment

There are several Zn^II^ complexes, with formula, [ZnCl_2_(N)_2_], such as [ZnCl_2_(AMS)_2_], (II) (Shen *et al.*, 2004), [ZnCl_2_(4-CNpy)_2_], (III) (Steffen & Palenik, 1977), [ZnCl_2_(pht)_2_], (IV) (Çelik *et al.*, 2004), [ZnCl_2_(quin)_2_], (V) (Cui *et al.*, 1998), [ZnCl_2_(quino)_2_], (VI) (Markowitz *et al.*, 2006) and [ZnCl_2_(meim)_2_], (VII) (Musie *et al.*, 2004) [where AMS is 3-Amino-5-methylisoxazole, 4-CNpy is 4-cyanopyridine, pht is phthalazine, quin is quinoline, quino is quinoxaline and meim is 1-methylimidazole] have been synthesized and characterized by single-crystal X-ray diffraction methods.

There are also several Zn^II^ complexes, with formula, [ZnCl_2_(N—N)], such as [ZnCl_2_(bipy)], (VIII) (Khan & Tuck, 1984), [ZnCl_2_(biim)], (IX) (Gruia *et al.*, 2007), [ZnCl_2_(phbipy)], (X) (Kozhevnikov *et al.*, 2006), [ZnCl_2_(phen)], (XI) (Reimann *et al.*, 1966), [ZnCl_2_(dmphen)], (XII) (Preston & Kennard, 1969), [ZnCl_2_(dpdmbip)], (XIII) (Liu *et al.*, 2004), [ZnCl_2_(dm4bt)], (XIV) (Khavasi *et al.*, 2008), [ZnCl_2_(5,5'-dmbpy)], (XV) (Khalighi *et al.*, 2008) and [ZnCl_2_(6-mbipy)], (XVI) (Ahmadi *et al.*, 2008) [where bipy is 2,2'-bipyridine, biim is 2,2'-biimidazole, phbipy is 5-phenyl-2,2'-bipyridine, phen is 1,10-phenanthroline, dmphen is 2,9-dimethyl-1,10-phenanthroline, dpdmbip is 4,4'-diphenyl-6,6'-dimethyl-2,2'-bipyrimidine, dm4bt is 2,2'-dimethyl-4,4'-bithiazole, 5,5'-dmbpy is 5,5'-dimethyl-2,2'-bipyridine and 6-mbipy is 6-methyl-2,2'-bipyridine] have been synthesized and characterized by single-crystal X-ray diffraction methods. We report herein the synthesis and crystal structure of the title compound, (I).

In the molecule of the title compound (Fig 1), Zn^II^ atom is four-coordinated in a distorted tetrahedral configuration by two N atoms from two phenanthridine and two terminal Cl atoms (Table 1). The bond lengths (Allen *et al.*, 1987) and angles are within normal ranges. Phenanthridine ring systems A (N1/C1-C13) and B (N2/C14-C26) are, of course, planar and the dihedral angle between them is A/B = 69.92 (3)°. Intramolecular C-H···Cl interaction (Table 2) results in the formation of a planar five-membered ring C (Zn1/Cl1/N1/C1/H1), which is oriented with respect to the adjacent phenanthridine ring system A at a dihedral angle of 8.32 (3)°.

In the crystal structure (Fig. 2), the π–π contacts between the phenanthridine rings, Cg2—Cg3^i^, Cg4—Cg6^ii^ and Cg6—Cg6^ii^, [symmetry codes: (i) 1 - x, -y, -z, (ii) -x, -y, -z, where Cg2, Cg3, Cg4 and Cg6 are centroids of the rings (C2-C7), (C8-C13), (N2/C14/C15/C20/C21/C26) and (C21-C26), respectively] may stabilize the structure, with centroid-centroid distances of 3.839 (2), 3.617 (1) and 3.682 (1) Å, respectively. There also exist two weak C—H···π interactions (Table 2).

### Experimental

For the preparation of the title compound, (I), a solution of phenanthridine (0.30 g, 1.66 mmol) in methanol (15 ml) was added to a solution of ZnCl_2_ (0.11 g, 0.83 mmol) in acetonitrile (30 ml) and the resulting colorless solution was stirred for 30 min at 313 K, and then it was left to evaporate slowly at room temperature. After one week, colorless prismatic crystals of the title compound were isolated (yield; 0.31 g, 75.5%).

### Refinement

H atoms were positioned geometrically, with C-H = 0.93 Å for aromatic H and constrained to ride on their parent atoms, with U_iso_(H) = 1.2U_eq_(C).

### Crystal data

**Table:**

[ZnCl_2_(C_13_H_9_N)_2_]	*F*(000) = 1008
*M**_r_* = 494.71	*D*_x_ = 1.543 Mg m^−^^3^
Monoclinic, *P*2_1_/*c*	Mo *K*α radiation, λ = 0.71073 Å
Hall symbol: -P 2ybc	Cell parameters from 1987 reflections
*a* = 16.193 (3) Å	θ = 2.5–29.3°
*b* = 10.101 (2) Å	µ = 1.42 mm^−^^1^
*c* = 14.491 (3) Å	*T* = 298 K
β = 116.02 (3)°	Prism, colorless
*V* = 2130.0 (9) Å^3^	0.45 × 0.30 × 0.22 mm
*Z* = 4	

### Data collection

**Table:**

Bruker SMART CCD area-detector diffractometer	5732 independent reflections
Radiation source: fine-focus sealed tube	4612 reflections with *I* > 2σ(*I*)
graphite	*R*_int_ = 0.041
φ and ω scans	θ_max_ = 29.3°, θ_min_ = 2.5°
Absorption correction: multi-scan (*SADABS*; Bruker, 1998)	*h* = −22→22
*T*_min_ = 0.610, *T*_max_ = 0.740	*k* = −13→13
16947 measured reflections	*l* = −19→19

### Refinement

**Table:**

Refinement on *F*^2^	Primary atom site location: structure-invariant direct methods
Least-squares matrix: full	Secondary atom site location: difference Fourier map
*R*[*F*^2^ > 2σ(*F*^2^)] = 0.037	Hydrogen site location: inferred from neighbouring sites
*wR*(*F*^2^) = 0.086	H-atom parameters constrained
*S* = 1.09	*w* = 1/[σ^2^(*F*_o_^2^) + (0.038*P*)^2^ + 0.5741*P*] where *P* = (*F*_o_^2^ + 2*F*_c_^2^)/3
5732 reflections	(Δ/σ)_max_ = 0.013
280 parameters	Δρ_max_ = 0.28 e Å^−^^3^
0 restraints	Δρ_min_ = −0.39 e Å^−^^3^

### Special details

**Table:**

Geometry. All e.s.d.'s (except the e.s.d. in the dihedral angle between two l.s. planes) are estimated using the full covariance matrix. The cell e.s.d.'s are taken into account individually in the estimation of e.s.d.'s in distances, angles and torsion angles; correlations between e.s.d.'s in cell parameters are only used when they are defined by crystal symmetry. An approximate (isotropic) treatment of cell e.s.d.'s is used for estimating e.s.d.'s involving l.s. planes.
Refinement. Refinement of *F*^2^ against ALL reflections. The weighted *R*-factor *wR* and goodness of fit *S* are based on *F*^2^, conventional *R*-factors *R* are based on *F*, with *F* set to zero for negative *F*^2^. The threshold expression of *F*^2^ > σ(*F*^2^) is used only for calculating *R*-factors(gt) *etc*. and is not relevant to the choice of reflections for refinement. *R*-factors based on *F*^2^ are statistically about twice as large as those based on *F*, and *R*- factors based on ALL data will be even larger.

### Fractional atomic coordinates and isotropic or equivalent isotropic displacement parameters (Å^2^)

**Table:**

	*x*	*y*	*z*	*U*_iso_*/*U*_eq_	
Zn1	0.259400 (15)	0.26111 (2)	0.120119 (17)	0.03590 (7)	
Cl1	0.25049 (4)	0.47913 (5)	0.09659 (5)	0.05264 (14)	
Cl2	0.27473 (4)	0.19233 (6)	0.27420 (4)	0.05102 (14)	
N1	0.37559 (11)	0.19993 (17)	0.10537 (13)	0.0373 (3)	
N2	0.14942 (10)	0.17454 (16)	−0.00248 (12)	0.0338 (3)	
C1	0.42820 (14)	0.2930 (2)	0.09724 (16)	0.0410 (4)	
H1	0.4056	0.3791	0.0863	0.049*	
C2	0.51762 (14)	0.2716 (2)	0.10399 (16)	0.0417 (4)	
C3	0.57131 (17)	0.3780 (3)	0.09923 (19)	0.0528 (6)	
H3	0.5481	0.4637	0.0902	0.063*	
C4	0.65812 (18)	0.3558 (3)	0.1079 (2)	0.0626 (7)	
H4	0.6936	0.4260	0.1039	0.075*	
C5	0.69249 (18)	0.2273 (3)	0.1228 (2)	0.0674 (8)	
H5	0.7512	0.2124	0.1283	0.081*	
C6	0.64215 (16)	0.1227 (3)	0.1293 (2)	0.0574 (6)	
H6	0.6672	0.0380	0.1401	0.069*	
C7	0.55249 (14)	0.1417 (2)	0.11974 (15)	0.0432 (5)	
C8	0.49487 (14)	0.0368 (2)	0.12662 (15)	0.0409 (4)	
C9	0.52121 (17)	−0.0978 (2)	0.13981 (17)	0.0503 (5)	
H9	0.5782	−0.1218	0.1441	0.060*	
C10	0.46418 (19)	−0.1931 (2)	0.14633 (18)	0.0548 (6)	
H10	0.4828	−0.2812	0.1547	0.066*	
C11	0.37861 (18)	−0.1601 (2)	0.14065 (18)	0.0517 (5)	
H11	0.3404	−0.2259	0.1453	0.062*	
C12	0.35073 (16)	−0.0305 (2)	0.12812 (16)	0.0448 (5)	
H12	0.2939	−0.0084	0.1251	0.054*	
C13	0.40733 (14)	0.0686 (2)	0.11980 (14)	0.0377 (4)	
C14	0.16296 (13)	0.12723 (19)	−0.07864 (15)	0.0360 (4)	
H14	0.2222	0.1316	−0.0736	0.043*	
C15	0.09352 (13)	0.06958 (18)	−0.16859 (14)	0.0348 (4)	
C16	0.11488 (16)	0.0160 (2)	−0.24580 (16)	0.0417 (4)	
H16	0.1747	0.0203	−0.2389	0.050*	
C17	0.04765 (18)	−0.0419 (2)	−0.33016 (18)	0.0486 (5)	
H17	0.0615	−0.0771	−0.3810	0.058*	
C18	−0.04216 (17)	−0.0482 (2)	−0.34024 (18)	0.0510 (5)	
H18	−0.0876	−0.0881	−0.3981	0.061*	
C19	−0.06479 (15)	0.0033 (2)	−0.26642 (17)	0.0454 (5)	
H19	−0.1250	−0.0022	−0.2746	0.054*	
C20	0.00307 (13)	0.06448 (18)	−0.17833 (14)	0.0343 (4)	
C21	−0.01463 (13)	0.12123 (18)	−0.09734 (15)	0.0343 (4)	
C22	−0.10280 (14)	0.1282 (2)	−0.10056 (17)	0.0430 (5)	
H22	−0.1531	0.0952	−0.1574	0.052*	
C23	−0.11556 (15)	0.1830 (2)	−0.02101 (19)	0.0481 (5)	
H23	−0.1743	0.1872	−0.0247	0.058*	
C24	−0.04179 (17)	0.2319 (2)	0.06441 (19)	0.0476 (5)	
H24	−0.0510	0.2678	0.1183	0.057*	
C25	0.04526 (15)	0.2277 (2)	0.07015 (17)	0.0417 (4)	
H25	0.0947	0.2608	0.1279	0.050*	
C26	0.05989 (12)	0.17376 (18)	−0.01046 (14)	0.0328 (4)	

### Atomic displacement parameters (Å^2^)

**Table:**

	*U*^11^	*U*^22^	*U*^33^	*U*^12^	*U*^13^	*U*^23^
Zn1	0.02853 (11)	0.03705 (12)	0.03880 (12)	−0.00174 (9)	0.01173 (8)	−0.00362 (9)
Cl1	0.0510 (3)	0.0376 (3)	0.0687 (4)	0.0014 (2)	0.0258 (3)	−0.0007 (2)
Cl2	0.0481 (3)	0.0596 (3)	0.0436 (3)	−0.0047 (3)	0.0185 (2)	0.0027 (2)
N1	0.0312 (8)	0.0388 (8)	0.0385 (8)	0.0034 (7)	0.0120 (7)	−0.0012 (7)
N2	0.0265 (7)	0.0339 (7)	0.0374 (8)	−0.0007 (6)	0.0107 (6)	−0.0010 (6)
C1	0.0341 (10)	0.0424 (10)	0.0442 (11)	0.0019 (8)	0.0149 (8)	−0.0014 (8)
C2	0.0317 (9)	0.0543 (12)	0.0367 (10)	−0.0003 (9)	0.0127 (8)	−0.0022 (9)
C3	0.0439 (12)	0.0640 (15)	0.0506 (13)	−0.0062 (11)	0.0207 (10)	−0.0017 (11)
C4	0.0429 (13)	0.087 (2)	0.0608 (15)	−0.0129 (13)	0.0250 (12)	−0.0031 (14)
C5	0.0333 (11)	0.104 (2)	0.0666 (16)	0.0047 (14)	0.0232 (11)	−0.0006 (15)
C6	0.0354 (11)	0.0780 (17)	0.0564 (14)	0.0117 (12)	0.0178 (10)	0.0020 (12)
C7	0.0311 (9)	0.0620 (13)	0.0325 (10)	0.0082 (9)	0.0103 (8)	−0.0010 (9)
C8	0.0339 (9)	0.0511 (11)	0.0306 (9)	0.0092 (9)	0.0075 (7)	−0.0005 (8)
C9	0.0453 (12)	0.0557 (13)	0.0419 (11)	0.0201 (11)	0.0116 (10)	0.0029 (10)
C10	0.0656 (16)	0.0453 (12)	0.0409 (11)	0.0180 (12)	0.0116 (11)	0.0035 (9)
C11	0.0556 (14)	0.0425 (11)	0.0462 (12)	−0.0001 (10)	0.0123 (10)	0.0037 (9)
C12	0.0395 (11)	0.0457 (11)	0.0430 (11)	0.0007 (9)	0.0125 (9)	−0.0004 (9)
C13	0.0329 (9)	0.0412 (10)	0.0317 (9)	0.0044 (8)	0.0074 (7)	−0.0023 (8)
C14	0.0286 (9)	0.0373 (9)	0.0394 (10)	−0.0009 (7)	0.0125 (8)	0.0010 (8)
C15	0.0346 (9)	0.0313 (9)	0.0351 (9)	−0.0016 (7)	0.0121 (8)	0.0008 (7)
C16	0.0436 (11)	0.0391 (10)	0.0431 (11)	0.0040 (9)	0.0198 (9)	0.0000 (8)
C17	0.0593 (14)	0.0412 (11)	0.0448 (12)	−0.0011 (10)	0.0223 (11)	−0.0077 (9)
C18	0.0519 (13)	0.0433 (11)	0.0459 (12)	−0.0085 (10)	0.0105 (10)	−0.0100 (9)
C19	0.0383 (10)	0.0408 (10)	0.0488 (12)	−0.0094 (9)	0.0116 (9)	−0.0039 (9)
C20	0.0313 (9)	0.0286 (8)	0.0375 (9)	−0.0026 (7)	0.0099 (7)	0.0025 (7)
C21	0.0306 (9)	0.0295 (8)	0.0405 (10)	−0.0020 (7)	0.0133 (8)	0.0036 (7)
C22	0.0307 (9)	0.0444 (11)	0.0513 (12)	−0.0046 (8)	0.0155 (9)	−0.0001 (9)
C23	0.0359 (10)	0.0465 (11)	0.0676 (15)	0.0004 (9)	0.0278 (10)	0.0024 (10)
C24	0.0468 (12)	0.0447 (11)	0.0588 (13)	0.0003 (10)	0.0302 (11)	−0.0060 (10)
C25	0.0378 (10)	0.0409 (10)	0.0455 (11)	−0.0024 (8)	0.0175 (9)	−0.0062 (8)
C26	0.0287 (8)	0.0290 (8)	0.0394 (9)	−0.0012 (7)	0.0138 (7)	0.0014 (7)

### Geometric parameters (Å, °)

**Table:**

Cl1—Zn1	2.2234 (7)	C12—H12	0.9300
Cl2—Zn1	2.2456 (7)	C13—N1	1.405 (3)
N1—Zn1	2.0785 (17)	C14—N2	1.306 (2)
N2—Zn1	2.0775 (17)	C14—C15	1.420 (3)
C1—N1	1.308 (3)	C14—H14	0.9300
C1—C2	1.424 (3)	C15—C20	1.410 (3)
C1—H1	0.9300	C15—C16	1.416 (3)
C2—C3	1.402 (3)	C16—C17	1.361 (3)
C2—C7	1.407 (3)	C16—H16	0.9300
C3—C4	1.374 (4)	C17—C18	1.398 (4)
C3—H3	0.9300	C17—H17	0.9300
C4—C5	1.392 (4)	C18—C19	1.376 (3)
C4—H4	0.9300	C18—H18	0.9300
C5—C6	1.363 (4)	C19—C20	1.410 (3)
C5—H5	0.9300	C19—H19	0.9300
C6—C7	1.410 (3)	C20—C21	1.443 (3)
C6—H6	0.9300	C21—C22	1.410 (3)
C7—C8	1.444 (3)	C21—C26	1.411 (3)
C8—C9	1.412 (3)	C22—C23	1.374 (3)
C8—C13	1.414 (3)	C22—H22	0.9300
C9—C10	1.366 (4)	C23—C24	1.380 (3)
C9—H9	0.9300	C23—H23	0.9300
C10—C11	1.392 (4)	C24—C25	1.376 (3)
C10—H10	0.9300	C24—H24	0.9300
C11—C12	1.371 (3)	C25—C26	1.400 (3)
C11—H11	0.9300	C25—H25	0.9300
C12—C13	1.398 (3)	C26—N2	1.403 (2)
			
N2—Zn1—N1	105.19 (7)	C11—C12—C13	120.3 (2)
N2—Zn1—Cl1	108.18 (5)	C11—C12—H12	119.8
N1—Zn1—Cl1	106.23 (5)	C13—C12—H12	119.8
N2—Zn1—Cl2	113.54 (5)	C12—C13—N1	118.57 (18)
N1—Zn1—Cl2	107.46 (6)	C12—C13—C8	120.43 (19)
Cl1—Zn1—Cl2	115.49 (3)	N1—C13—C8	121.00 (19)
C1—N1—C13	118.80 (18)	N2—C14—C15	124.52 (18)
C1—N1—Zn1	116.76 (14)	N2—C14—H14	117.7
C13—N1—Zn1	123.65 (14)	C15—C14—H14	117.7
C14—N2—C26	118.60 (16)	C20—C15—C16	120.69 (18)
C14—N2—Zn1	118.54 (13)	C20—C15—C14	118.46 (18)
C26—N2—Zn1	122.69 (13)	C16—C15—C14	120.84 (19)
N1—C1—C2	124.6 (2)	C17—C16—C15	119.8 (2)
N1—C1—H1	117.7	C17—C16—H16	120.1
C2—C1—H1	117.7	C15—C16—H16	120.1
C3—C2—C7	120.6 (2)	C16—C17—C18	119.9 (2)
C3—C2—C1	120.9 (2)	C16—C17—H17	120.0
C7—C2—C1	118.4 (2)	C18—C17—H17	120.0
C4—C3—C2	120.2 (3)	C19—C18—C17	121.4 (2)
C4—C3—H3	119.9	C19—C18—H18	119.3
C2—C3—H3	119.9	C17—C18—H18	119.3
C3—C4—C5	119.3 (3)	C18—C19—C20	120.1 (2)
C3—C4—H4	120.3	C18—C19—H19	119.9
C5—C4—H4	120.3	C20—C19—H19	119.9
C6—C5—C4	121.5 (2)	C15—C20—C19	118.01 (19)
C6—C5—H5	119.2	C15—C20—C21	118.19 (17)
C4—C5—H5	119.2	C19—C20—C21	123.80 (18)
C5—C6—C7	120.6 (3)	C22—C21—C26	117.92 (18)
C5—C6—H6	119.7	C22—C21—C20	123.54 (18)
C7—C6—H6	119.7	C26—C21—C20	118.54 (17)
C2—C7—C6	117.8 (2)	C23—C22—C21	121.0 (2)
C2—C7—C8	118.03 (19)	C23—C22—H22	119.5
C6—C7—C8	124.2 (2)	C21—C22—H22	119.5
C9—C8—C13	117.5 (2)	C22—C23—C24	120.5 (2)
C9—C8—C7	123.3 (2)	C22—C23—H23	119.8
C13—C8—C7	119.14 (19)	C24—C23—H23	119.8
C10—C9—C8	121.0 (2)	C25—C24—C23	120.2 (2)
C10—C9—H9	119.5	C25—C24—H24	119.9
C8—C9—H9	119.5	C23—C24—H24	119.9
C9—C10—C11	120.9 (2)	C24—C25—C26	120.4 (2)
C9—C10—H10	119.6	C24—C25—H25	119.8
C11—C10—H10	119.6	C26—C25—H25	119.8
C12—C11—C10	119.8 (2)	C25—C26—N2	118.43 (17)
C12—C11—H11	120.1	C25—C26—C21	119.96 (18)
C10—C11—H11	120.1	N2—C26—C21	121.61 (17)
			
N1—C1—C2—C3	−177.0 (2)	C18—C19—C20—C21	179.9 (2)
N1—C1—C2—C7	0.5 (3)	C15—C20—C21—C22	176.71 (18)
C7—C2—C3—C4	1.3 (3)	C19—C20—C21—C22	−3.7 (3)
C1—C2—C3—C4	178.7 (2)	C15—C20—C21—C26	−2.7 (3)
C2—C3—C4—C5	−0.8 (4)	C19—C20—C21—C26	176.82 (19)
C3—C4—C5—C6	−0.2 (4)	C26—C21—C22—C23	−0.8 (3)
C4—C5—C6—C7	0.8 (4)	C20—C21—C22—C23	179.8 (2)
C3—C2—C7—C6	−0.7 (3)	C21—C22—C23—C24	−0.4 (3)
C1—C2—C7—C6	−178.2 (2)	C22—C23—C24—C25	0.8 (4)
C3—C2—C7—C8	178.41 (19)	C23—C24—C25—C26	0.0 (3)
C1—C2—C7—C8	0.9 (3)	C24—C25—C26—N2	178.40 (19)
C5—C6—C7—C2	−0.4 (4)	C24—C25—C26—C21	−1.2 (3)
C5—C6—C7—C8	−179.4 (2)	C22—C21—C26—C25	1.5 (3)
C2—C7—C8—C9	178.4 (2)	C20—C21—C26—C25	−179.01 (17)
C6—C7—C8—C9	−2.5 (3)	C22—C21—C26—N2	−178.02 (17)
C2—C7—C8—C13	−1.5 (3)	C20—C21—C26—N2	1.4 (3)
C6—C7—C8—C13	177.6 (2)	C2—C1—N1—C13	−1.3 (3)
C13—C8—C9—C10	−0.4 (3)	C2—C1—N1—Zn1	168.86 (16)
C7—C8—C9—C10	179.7 (2)	C12—C13—N1—C1	−179.80 (18)
C8—C9—C10—C11	−0.3 (4)	C8—C13—N1—C1	0.7 (3)
C9—C10—C11—C12	0.2 (4)	C12—C13—N1—Zn1	10.7 (2)
C10—C11—C12—C13	0.8 (3)	C8—C13—N1—Zn1	−168.77 (14)
C11—C12—C13—N1	179.0 (2)	C15—C14—N2—C26	−2.6 (3)
C11—C12—C13—C8	−1.5 (3)	C15—C14—N2—Zn1	−177.82 (14)
C9—C8—C13—C12	1.3 (3)	C25—C26—N2—C14	−178.37 (18)
C7—C8—C13—C12	−178.80 (18)	C21—C26—N2—C14	1.2 (3)
C9—C8—C13—N1	−179.20 (18)	C25—C26—N2—Zn1	−3.3 (2)
C7—C8—C13—N1	0.7 (3)	C21—C26—N2—Zn1	176.23 (13)
N2—C14—C15—C20	1.2 (3)	C14—N2—Zn1—N1	−15.81 (16)
N2—C14—C15—C16	−177.53 (18)	C26—N2—Zn1—N1	169.13 (14)
C20—C15—C16—C17	−0.4 (3)	C14—N2—Zn1—Cl1	97.39 (14)
C14—C15—C16—C17	178.30 (19)	C26—N2—Zn1—Cl1	−77.67 (14)
C15—C16—C17—C18	0.0 (3)	C14—N2—Zn1—Cl2	−133.03 (13)
C16—C17—C18—C19	0.2 (4)	C26—N2—Zn1—Cl2	51.91 (15)
C17—C18—C19—C20	0.1 (3)	C1—N1—Zn1—N2	121.89 (15)
C16—C15—C20—C19	0.7 (3)	C13—N1—Zn1—N2	−68.46 (16)
C14—C15—C20—C19	−178.05 (18)	C1—N1—Zn1—Cl1	7.32 (16)
C16—C15—C20—C21	−179.75 (17)	C13—N1—Zn1—Cl1	176.97 (14)
C14—C15—C20—C21	1.5 (3)	C1—N1—Zn1—Cl2	−116.83 (14)
C18—C19—C20—C15	−0.6 (3)	C13—N1—Zn1—Cl2	52.82 (15)

### Hydrogen-bond geometry (Å, °)

**Table:**

*D*—H···*A*	*D*—H	H···*A*	*D*···*A*	*D*—H···*A*
C1—H1···Cl1	0.93	2.77	3.434 (3)	129
C17—H17···Cg6^i^	0.93	2.82	3.535 (3)	134
C24—H24···Cg5^ii^	0.93	2.81	3.508 (3)	132

Symmetry codes: (i) −*x*, *y*−1/2, −*z*−1/2; (ii) *x*, −*y*−1/2, *z*−1/2.
